# Mechanism-Informed Interfacial Chemistry and Structural Evolution of TiS_2_ During Ca^2+^ Intercalation in Concentrated Aqueous CaCl_2_ Electrolytes

**DOI:** 10.3390/ijms262411971

**Published:** 2025-12-12

**Authors:** SangYup Lee, Sujin Seong, Seunga Yang, Soon-Ki Jeong

**Affiliations:** 1 Department of Future Convergence Technology, Graduate School, Soonchunhyang University, Soonchunhyang-ro 22-gil, Sinchang-myeon, Asan-si 31538, Chungcheongnam-do, Republic of Korea; 20237450@sch.ac.kr (S.L.); sujin@sch.ac.kr (S.S.); tmddk1107@sch.ac.kr (S.Y.); 2 Department of Energy Engineering, Soonchunhyang University, Soonchunhyang-ro 22-gil, Sinchang-myeon, Asan-si 31538, Chungcheongnam-do, Republic of Korea; 3 Advanced Energy Research Center, Soonchunhyang University, Soonchunhyang-ro 22-gil, Sinchang-myeon, Asan-si 31538, Chungcheongnam-do, Republic of Korea

**Keywords:** TiS_2_, calcium-ion battery, aqueous electrolyte, interfacial chemistry, structural evolution, potential window

## Abstract

This study examines the interfacial and structural evolution of titanium disulfide (TiS_2_) during Ca^2+^ intercalation/deintercalation in concentrated aqueous CaCl_2_. Electrochemical measurements were combined with ex situ X-ray diffraction (XRD), X-ray photoelectron spectroscopy (XPS), and Raman spectroscopy to characterize the solvation structure, potential window, and reversibility in concentrated CaCl_2_ electrolytes. Increasing the CaCl_2_ concentration from 1.0 to 8.0 M was accompanied by reduced gas evolution and an expanded practical operating window. Stepwise analysis identified the potential range −1.00 to 0.10 V (vs. the saturated calomel electrode) as a practical window that minimized TiO_2_/S_8_ formation while preserving reversible Ca^2+^ intercalation. Ex situ XRD showed reversible (001) shifts, consistent with interlayer expansion and contraction, and peak broadening was indicative of partial amorphization and defects. XPS revealed CaS and polysulfides (S_z_^2−^, 2 ≤ z ≤ 8) to be the prevalent surface species with limited Ca(OH)_2_ and CaSO_4_; within the detection limits, no chlorine-containing reduction products were observed after charging. The electrochemical and spectroscopic results indicate that intercalation is accompanied by partial sulfur-centered reduction and defect signatures, with associated changes in the interfacial charge-transfer characteristics and reversibility. These findings link the potential, interfacial chemistry, and lattice response, and suggest design considerations for stable aqueous multivalent-ion storage.

## 1. Introduction

Lithium-ion batteries currently dominate the rechargeable energy-storage market owing to their high energy density and technological maturity. However, concerns regarding resource distribution, cost, and safety under abusive conditions motivate the exploration of complementary chemistries, including aqueous systems. Rechargeable aqueous multivalent-ion batteries have been explored as safe and sustainable complements to lithium-ion technology [1,2,3], with recent reviews consolidating the status, challenges, and positive electrode/host design principles for multivalent systems [4,5,6]. However, in aqueous media, water electrolysis, most notably the hydrogen evolution reaction (HER) and parasitic interfacial reactions, often constrain the practical operating window more than the intrinsic properties of the host and this adversely affects the durability and efficiency [7,8,9]. Consequently, electrolyte structure control, particularly in highly concentrated aqueous electrolytes intended to lower the free-water activity and strengthen ion pairing, has been adopted to steer the reaction selectivity toward solid-state storage pathways. This motivated concentration–potential–interfacial/structural analyses that explicitly link the electrolyte environment to interfacial speciation, charge-transfer, and structural reversibility in multivalent hosts [8,9,10]. Within this landscape, aqueous calcium ion (Ca^2+^) systems are appealing because of the elemental abundance of Ca and low standard reduction potential of Ca^2+^, but these systems are disadvantaged by their slow solid-state diffusion, strong electrostatic interactions with host lattices, and surface-side reactions [11,12,13]. Accordingly, highly concentrated aqueous electrolytes offer tunable solvation and electric double-layer structures to extend the usable window and moderate parasitic reactions. This prompted the use of a platform electrolyte that enables the concentration to be controlled over a wide range and spectroscopically resolvable solvation changes to investigate the concentration–potential effects in Ca^2+^ cells [14,15,16].

Among the candidate salts, calcium chloride (CaCl_2_) offers such a platform: its high solubility under ambient conditions supports the stable preparation of concentrated solutions across a wide concentration range, which allows the water activity to be systematically tuned and ion pairing without additives [14,15,16]. In concentrated CaCl_2_ solutions, the Ca^2+^ solvation environment evolves from solvent-separated ion pairs to contact ion pairs; in addition, the reduced free-water population can be resolved spectroscopically, which provides a direct means to link the solvation structure to the electrochemical behavior [14,15,16]. Concentrated environments can also alter the reaction selectivity—for example, by modifying the relative propensity for anion-involved pathways (e.g., partial anion reduction)—which reinforces the need for concentration- and potential-resolved interrogation in CaCl_2_ media [14,15,16].

Under these circumstances, layered titanium disulfide (TiS_2_) serves as a suitable model host because its two-dimensional framework of edge-sharing TiS_6_ octahedra and the variable formal oxidation state of titanium support multivalent intercalation in a structurally well-defined lattice. TiS_2_ has also been widely investigated as a cathode in nonaqueous Li^+^, Na^+^, Mg^2+^, and Ca^2+^ systems, providing a useful benchmark for multivalent intercalation behavior in layered sulfides. Compared with other layered disulfides such as MoS_2_ or VS_2_, TiS_2_ has more frequently been employed as a reference material for multivalent-ion storage, which makes it a convenient platform for establishing concentration–potential–structure correlations in aqueous CaCl_2_ electrolytes. In aqueous media, however, oxidative side products (e.g., TiO_2_ and S_8_) can form when the potential deviates from the favorable range, which undermines the reversibility [10,15,16]. Despite reports on the storage of aqueous Ca^2+^ in TiS_2_, the combined influence of the CaCl_2_ concentration and applied potential on interfacial speciation and structural response has not been clarified [11]. In previous work, TiS_2_ was demonstrated as an active material for aqueous Ca-ion batteries in both CaCl_2_ and Ca(NO_3_)_2_ electrolytes, with emphasis on overall capacity, cycling behavior, and anion-dependent performance [15]. In contrast, the present study focuses on concentrated CaCl_2_ as a platform electrolyte to elucidate how the CaCl_2_ concentration and applied potential jointly govern interfacial speciation, lattice response, and reversibility. To this end, potential-resolved electrochemistry is combined with ex situ X-ray diffraction (XRD), X-ray photoelectron spectroscopy (XPS), and Raman spectroscopy to construct a mechanism-oriented concentration–potential–response framework that extends the earlier performance-oriented study into a more detailed mechanistic analysis. This framework formally establishes a practical potential window by distinguishing regimes dominated by intercalation from those dominated by decomposition and is subsequently used to analyze interfacial and solvation differences between concentrated and dilute CaCl_2_ electrolytes, thereby rationalizing the conditions under which Ca^2+^ intercalation is reversible.

## 2. Results and Discussion

### 2.1. Electrochemical Behavior of TiS_2_ in Different CaCl_2_ Concentrations

**Figure 1:**
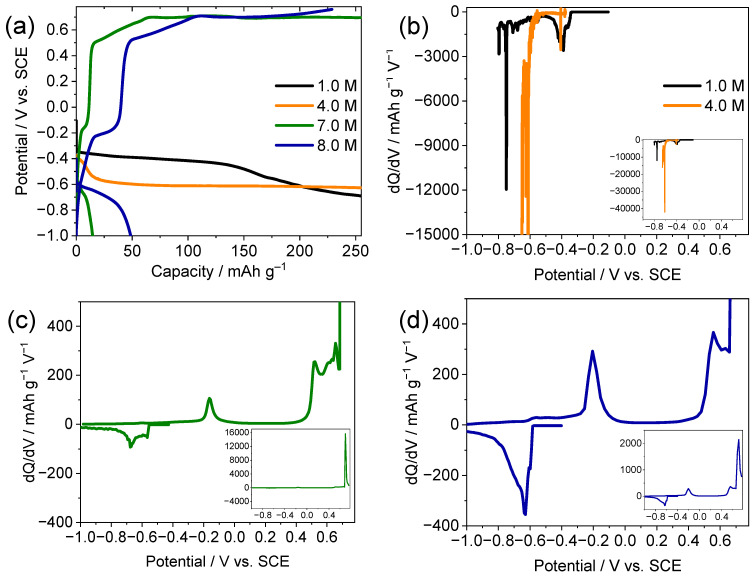
Electrochemical performance of TiS_2_ electrodes in aqueous CaCl_2_ electrolytes of different concentrations. (**a**) First-cycle galvanostatic charge–discharge profiles and (**b**–**d**) corresponding dQ/dV curves in electrolytes of (**b**) 1.0, 4.0, (**c**) 7.0, and (**d**) 8.0 M CaCl_2_.

The electrochemical behavior of the TiS_2_ electrodes was systematically investigated in aqueous CaCl_2_ electrolytes in the concentration range of 1.0 to 8.0 M. As shown in Figure 1a, the galvanostatic charge–discharge profiles of the first cycle display sloping regions with subtle inflections near −0.40 and −0.60 V vs. the saturated calomel electrode (SCE) in low concentrations of the electrolyte (1.0 and 4.0 M). The capacity increase was accompanied by visible gas evolution, which is consistent with hydrogen evolution under dilute conditions [8,9]. The differential capacity (dQ/dV) curves of these low-concentration cells (Figure 1b) exhibit broad asymmetric peaks without clear redox pairing, suggesting that the contribution from electrolyte decomposition is non-trivial relative to reversible Ca^2+^ intercalation.

At higher electrolyte concentrations of 7.0 and 8.0 M, the electrochemical responses changed markedly (Figure 1c,d). Distinct redox plateaus emerged during both charging and discharging. The potential decreased gradually to −1.00 V during charging, and the corresponding first-charge capacities reached 15 mAh g^−1^ for 7.0 M and 49 mAh g^−1^ for 8.0 M CaCl_2_ solutions. The markedly larger first-charge capacity in 8.0 M is therefore interpreted as arising from the extended practical potential window and more favorable interfacial environment, which enable deeper Ca^2+^ intercalation with reduced parasitic reactions, rather than from the onset of a distinct reaction mechanism compared with 7.0 M. These well-defined plateaus and the reduced polarization are consistent with the reduced HER contribution in the 8.0 M electrolyte, where Ca^2+^ intercalation signatures became more prominent within a wider practical potential window. During discharge, the stable plateau that appeared near −0.20 V was attributed to Ca^2+^ deintercalation. The small secondary plateau near 0.40 V may reflect oxidative side processes (e.g., water/adsorbate oxidation) and limited electrolyte decomposition. When the potential window is extended beyond −1.00 V, the initial cycles in 8.0 M CaCl_2_ lead to significant conversion reactions (formation of CaS, polysulfides, and minor Ca(OH)_2_/CaSO_4_), which contribute to a large irreversible capacity; this observation motivated the use of the narrower practical window of −1.00 to 0.10 V vs. SCE, within which the coulombic efficiency rapidly approaches ~95–100% and the capacity retention over 50 cycles remains moderate but stable.

The progression from dilute to concentrated CaCl_2_ electrolytes revealed a transition in the dominant reaction regime from behavior dominated by electrolyte decomposition (e.g., HER) to a regime where intercalation becomes the dominant contribution within a mixed intercalation–conversion response. Increasing the salt concentration was consistent with a reorganization of the electric double layer, accompanied by a more cation-rich interfacial environment, enhanced Ca^2+^-intercalation signatures, and visibly reduced gas evolution. Taken together, these observations are consistent with a qualitatively reduced contribution from parasitic hydrogen evolution in the concentrated electrolytes, although direct gas-quantification measurements were not performed in this study. This shift reflects substantial reorganization of the interfacial environment, in which decreased free-water activity and the formation of solvent-separated ion pairs or contact ion pairs modulate the electrochemical stability of the aqueous system [8,9,10,17]. Accordingly, the 8.0 M CaCl_2_ electrolyte offered a wider practical potential window, which enables reversible Ca^2+^ intercalation in TiS_2_ within ranges not observed under the dilute conditions tested here. Additional rate-capability measurements for the TiS_2_ electrode in 8.0 M CaCl_2_ within the tested C-rate range (0.1–5.0 C) are provided in the Supporting Information (Figure S1); the discharge capacity decreases monotonically with increasing C-rate but nearly recovers to its initial value when the current density is returned to 0.1 C, indicating reversible Ca^2+^ intercalation with kinetically limited behavior at high rates. Furthermore, extended cycling at 0.1 C within the same potential window (Figure S2) shows gradual capacity fading accompanied by coulombic efficiencies approaching ~95–100%, which is consistent with a qualitatively reduced contribution from parasitic reactions in the 8.0 M electrolyte.

### 2.2. Raman Analysis of Solvation Structure in CaCl_2_ Electrolytes

**Figure 2:**
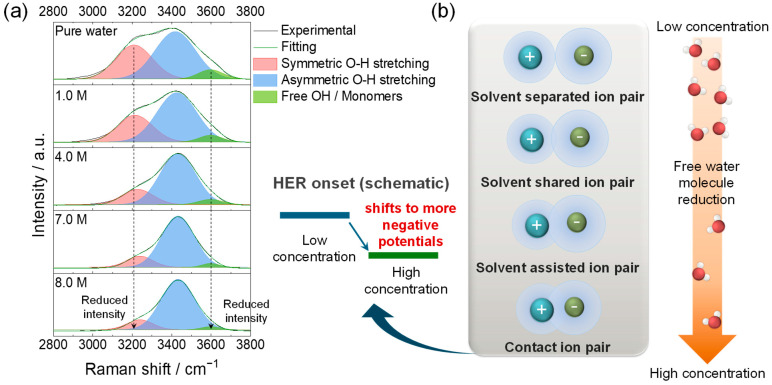
(**a**) Raman spectra of the O–H stretching region (2800–4000 cm^−1^) for pure water and CaCl_2_ solutions of 1.0, 4.0, 7.0, and 8.0 M. (**b**) Schematic linking the evolution of the O–H band (**left**), the concentration-dependent ion-pair topology (**right**; SSIP → SIP → CIP), and the shift in the apparent HER onset to more negative potentials with increasing CaCl_2_ concentration (**center**); conceptual illustration, not to scale.

Raman spectral bands in the O–H stretching region (2800–4000 cm^−1^) revealed the concentration-dependent evolution of hydrogen-bonding and Ca–Cl association, which provided solvation descriptors relevant to the observed regime transition. The three characteristic bands that appeared in the O–H stretching region near 3200, 3420, and 3600 cm^−1^ (Figure 2a) are commonly associated with strongly hydrogen-bonded (~3200 cm^−1^), moderately hydrogen-bonded (~3420 cm^−1^), and weakly hydrogen-bonded/‘free’ O–H (~3600 cm^−1^) components, respectively [18,19]. At low concentrations (1.0 and 4.0 M), the dominating features at 3200 and 3420 cm^−1^ indicated extended hydrogen-bonded networks.

As the CaCl_2_ concentration increased up to 7.0 and 8.0 M, the intensity of the symmetric O–H band at ~3200 cm^−1^ decreased, while the main O–H stretching component near 3420 cm^−1^ underwent a blue shift. Quantitatively, the band maximum shifted from 3420 cm^−1^ for pure water to 3433 cm^−1^ for the 8.0 M CaCl_2_ solution, corresponding to a net blue shift of ~13 cm^−1^. Deconvolution of the O–H stretching envelope showed that the normalized area ratio A_3200_/A_3420_ decreased from 0.63 to 0.16, and the ratio A_3600_/A_3420_ decreased from 0.17 to 0.04 between pure water and the 8.0 M CaCl_2_ solution (Table S1). These changes indicate a progressive reduction in bulk-like and ‘free’ water and stronger Ca^2+^–H_2_O interactions in the highly concentrated CaCl_2_ electrolytes, consistent with previous studies on concentrated aqueous electrolytes and water-in-salt systems. These spectral changes are consistent with the reduced population of free water and strengthened cation–anion interactions that perturb the hydrogen-bonded network [20]. The blue-shift in the ~3420 cm^−1^ component is consistent with a stronger local electric field around Ca^2+^ and reduced dielectric screening, suggesting more strongly coordinated and more compact hydration shells.

These changes mark a transition in the Ca^2+^ solvation environment from solvent-separated ion pairs (SSIP) at low concentrations to solvent-shared ion pairs (SIP), solvent-assisted ion pairs (SAIP), and contact ion pairs (CIP) at higher concentrations [18,19]. These terms describe the progressive shortening of the Ca–Cl distance and more limited participation of water in the first solvation shell. Beyond 7.0 M, the electrolyte entered a more ion-associated regime with enhanced Ca–Cl coordination (SSIP → SIP → SAIP → CIP), as illustrated schematically in Figure 2b.

Considered together with the electrochemical observations, the spectral evolution is consistent with lower free-water activity and strengthened cation–anion association and is consistent with a qualitative shift in the apparent HER onset to more negative potentials and an expanded practical potential window [21]. Overall, high-concentration CaCl_2_ electrolytes exhibit greater electrochemical stability [22], consistent with the lower free-water activity and strengthened Ca–Cl coordination. In comparison, the dilute regime (1.0–4.0 M) is characterized by O–H features indicative of extended hydrogen-bond networks, whereas the spectral results for the concentrated regime (7.0–8.0 M) point to lower free-water activity and enhanced Ca–Cl association; these differences in solvation are used below to rationalize the conditions under which reversible Ca^2+^ intercalation becomes accessible. These solvation changes are also consistent with the more compact hydration shells at higher concentrations, suggesting a reduced desolvation penalty at the interface, which could contribute to the greater accessibility of reversible Ca^2+^ intercalation in concentrated CaCl_2_ [23]. The Raman analysis in this study is not intended to introduce a new spectroscopic methodology for CaCl_2_ solutions; rather, it serves to provide system-specific solvation descriptors (changes in O–H band shape and position at fixed molarity) that can be directly correlated with the electrochemical regime transition and the emergence of reversible Ca^2+^ intercalation in TiS_2_.

### 2.3. Potential-Window-Resolved CV and Reaction Assignment

**Figure 3:**
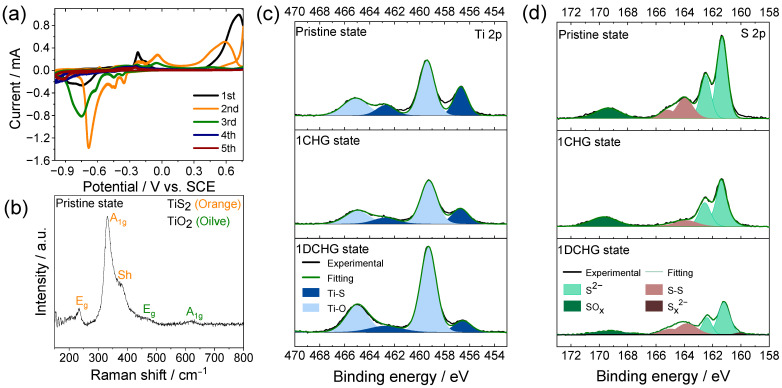
(**a**) Cyclic voltammograms of the TiS_2_ electrode in 8.0 M CaCl_2_ electrolyte solution. (**b**) Raman spectrum of the pristine TiS_2_ electrode. XPS (**c**) Ti 2p and (**d**) S 2p spectra of the TiS_2_ electrode in the pristine, first-charged (1CHG), and first-discharged (1DCHG) states.

The redox behavior of TiS_2_ in the 8 M CaCl_2_ electrolyte was examined using CV to resolve the potential-dependent reaction processes [24]. As shown in Figure 3a, several reduction and oxidation peaks appeared between −1.00 and 0.76 V vs. the saturated calomel electrode (SCE). The dominant reduction peak near −0.72 V was assigned to sulfur-centered reduction, which leads to the formation of polysulfide (S_z_^2−^) or HS^−^-type species at the interface, whereas the smaller peak at −0.24 V is tentatively assigned to a Ti^4+^/Ti^3+^ redox contribution [24,25,26,27]. The oxidation features above −0.20 V were consistent with the re-oxidation of reduced sulfur species, indicating the participation of both titanium and sulfur in distinct potential domains. The coexistence of these redox pairs is consistent with a mixed intercalation–conversion response [25,28,29,30,31,32], in which coupling between the Ti and S centers may contribute to charge compensation and stepwise reaction profiles [29]. In the first charge over the wider potential window, the CV suggests that Ca^2+^ intercalation into TiS_2_ is followed rapidly by conversion-type reactions at more negative potentials, so that any transient Ti^3+^ species formed during intercalation are likely to be quickly consumed as the system proceeds into the sulfur-centered conversion regime [33].

The Raman spectra of pristine TiS_2_ established vibrational benchmarks that were used to identify the redox-induced features in the subsequent spectra. The Raman spectrum in Figure 3b showed two fundamental vibrational modes of TiS_2_ (E_g_ at 236 cm^−1^ and A_1g_ at 320 cm^−1^), together with a weak shoulder near 370 cm^−1^ that is often attributed to defect-/disorder-activated features. These defect-related signatures are consistent with a slight sulfur deficiency and local modulation of the Ti valence, with implications for local electronic structures [28,34,35]. A weak band at ~616 cm^−1^ was consistent with the A_1g_ mode of rutile TiO_2_, while the feature near ~447 cm^−1^ corresponded to the E_g_ mode; together these observations indicate that the surface of TiS_2_ had been partially oxidized.

The XPS profiles (Figure 3c,d) corroborated these observations. The pristine electrode contained Ti–S and S–S components, whereas the oxide-related Ti–O and S–O (SO_x_) components observed after the first cycle indicated surface redox/oxidation changes [34]. These observations are consistent with the initial conditioning process in which a self-formed interphase was proposed to facilitate interfacial charge-transfer under concentrated CaCl_2_ conditions [36]. Here, the self-formed interphase denotes a reaction-derived surface layer observed ex situ after the first cycle. This layer comprised CaS and polysulfides as the prevalent species, with minor amounts of Ca(OH)_2_/CaSO_4_ and no chlorine-containing reduction products within the XPS detection limits of our measurements (Figure 3c,d).

**Figure 4:**
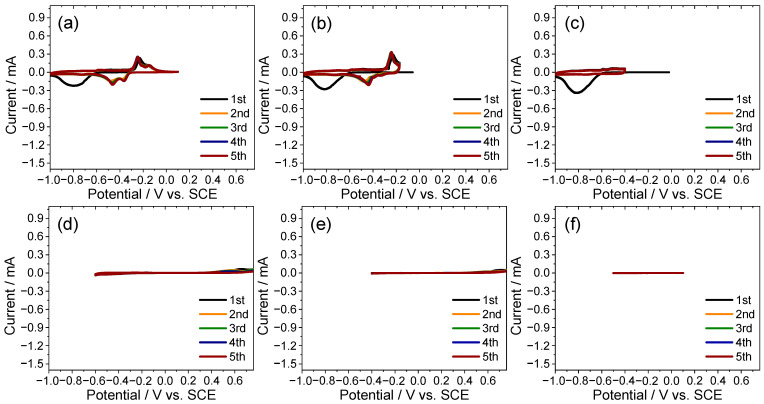
Potential-resolved CV for identifying redox pairs and delineating the practical potential window of the TiS_2_ electrode in 8.0 M CaCl_2_ electrolyte. (**a**) −1.00 to 0.10 V, (**b**) −1.00 to −0.17 V, (**c**) −1.00 to −0.40 V, (**d**) −0.60 to 0.76 V, (**e**) −0.40 to 0.76 V, and (**f**) −0.50 to 0.10 V.

To further separate the reversible and irreversible responses, stepwise CV scans were performed over negative and restricted windows. In the range −1.00 to 0.10 V (Figure 4a–c) large irreversible currents were suppressed and the redox pairs revealed at −0.72/−0.59 V and −0.48/−0.24 V were tentatively assigned to sulfur- and titanium-centered couples, respectively. By contrast, windows that did not extend beyond −0.60 V (Figure 4d–f) showed markedly reduced currents and diminished sulfur-related features, indicating that the −1.00 to −0.60 V region hosts the primary sulfur-centered response and defining −1.00 to 0.10 V as a practical operating range under concentrated CaCl_2_ conditions.

Based on these analyses, the potential window of −1.00 to 0.10 V was operationally identified as a practical range in which the intercalation activity is balanced with electrolyte stability, with minimized parasitic features. In subsequent investigations (Section 2.4), this window was used to differentiate behavior dominated by intercalation from that dominated by electrolyte decomposition (e.g., HER) under concentrated CaCl_2_ conditions.

### 2.4. Stepwise Structural Evolution Analyzed by Ex Situ XRD

**Figure 5:**
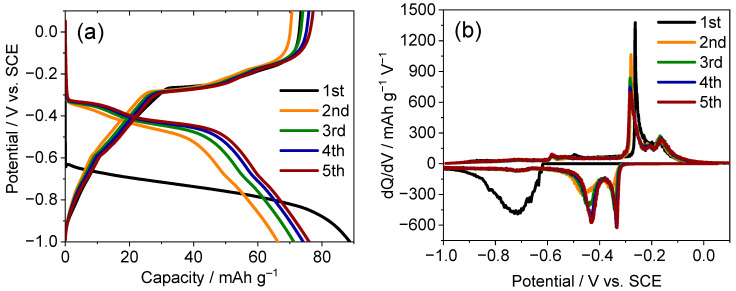
Electrochemical performance of the TiS_2_ electrode in 8.0 M CaCl_2_ within the practical potential window (−1.00 to 0.10 V): (**a**) Galvanostatic charge–discharge profiles showing stepwise plateaus and (**b**) corresponding dQ/dV curves exhibiting reproducible redox pairs over multiple cycles.

**Figure 6:**
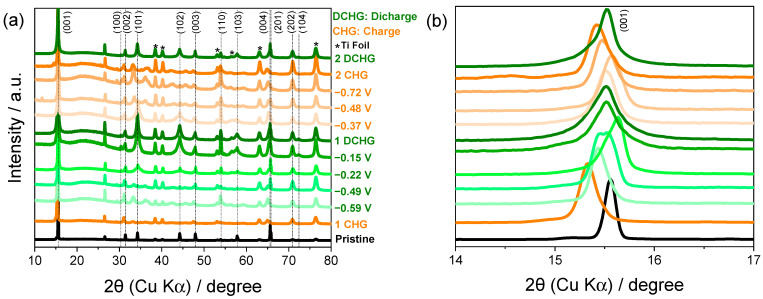
(**a**) Ex situ XRD patterns showing the structural evolution of TiS_2_ during galvanostatic cycling in 8.0 M CaCl_2_ and (**b**) magnified view of the (001) reflection to reveal reversible interlayer expansion and contraction.

Within the practical potential window (−1.00 to 0.10 V vs. SCE) established in Section 2.3, the electrochemical response of TiS_2_ in 8.0 M CaCl_2_ was examined by recording the galvanostatic profiles and conducting dQ/dV analyses (Figure 5). The charge curve exhibits three plateaus at approximately −0.72, −0.48, and −0.37 V with corresponding discharge features at −0.59, −0.24, and −0.15 V (Figure 5a), while the dQ/dV traces (Figure 5b) resolve matched redox pairs that coincide with the couples identified with CV in Section 2.3 [37,38]. These signatures are consistent with multistep configurational changes of Ca_x_TiS_2_ rather than a single continuous solid-solution process and delineate plateau-selected states for structural interrogation [39]. Accordingly, ex situ XRD was performed at representative states to correlate each electrochemical segment with the lattice evolution. The following paragraphs (Figure 6a,b; Table 1) detail the reversible (001) breathing and transient peak changes that mirror the plateau sequence.

The ex situ XRD patterns in Figure 6a reveal systematic variations in the peak intensity and position during cycling. During charging, the reflections of the (101), (102), and (103) planes gradually diminished, while transient reflections appeared between 30° and 50° (2θ). These reflections vanished upon discharge and the original peaks reappeared, indicating a quasi-reversible, multistep structural evolution. The reversible shift in the (001) reflection toward lower 2θ angles during charge and its return upon discharge (Figure 6b) is consistent with interlayer expansion during Ca^2+^ intercalation and contraction during deintercalation [31,40]. These observations are consistent with the coupling between ion insertion and lattice relaxation; the voltage plateaus correlate with the distinct configurations of the Ca_x_TiS_2_ lattice. Notably, the most pronounced shift in the (001) reflection is observed for the sample stopped at −0.22 V, where the cathodic current in the CV is already small. This shift is interpreted as structural relaxation and partial Ca^2+^ deintercalation that occur after the main sulfur-centered reduction at more negative potentials (−1.00 to −0.60 V), rather than as the onset of a separate redox process. This interpretation is consistent with the electrochemical features observed in the CV curves. In addition to the Ca_x_TiS_2_ reflections, no extra peaks attributable to crystalline TiO_2_ or S_8_ were detected within the sensitivity and angular resolution of the laboratory diffractometer, suggesting that the oxide and sulfur species identified by XPS are confined to thin, poorly crystalline surface layers rather than forming bulk transformation products of TiS_2_.

Quantitative analysis based on Bragg’s law (Table 1) showed that the interlayer spacing increased from 5.69 Å (pristine) to 5.77 Å after charging and returned to 5.71 Å after discharge [41] (Δd_001_ ~ +0.08 Å on charge; net change vs. pristine ~ +0.02 Å). The reversible recovery of spacing is consistent with a reversible lattice response that accommodates distortion without collapse. The intercalated Ca^2+^ may screen charge and polarize the Ti–S framework, which would facilitate modulation of the local lattice strain [35]; the transport metrics were not measured here. The observed broadening of the XRD peaks after cycling is consistent with partial amorphization and defect formation (e.g., sulfur vacancies), which can modulate local electronic pathways and may introduce limited charge localization [42].

Overall, TiS_2_ exhibited quasi-reversible structural states during the intercalation/deintercalation of Ca^2+^ within the tested range. Under these conditions, the layered framework is able to accommodate distortion without collapsing. To identify the chemical origin of these reversible lattice shifts and residual disorders, the surface speciation was analyzed using XPS across the same potential segments.

### 2.5. Surface Reaction Products and Interfacial Chemistry from XPS Analysis

**Figure 7:**
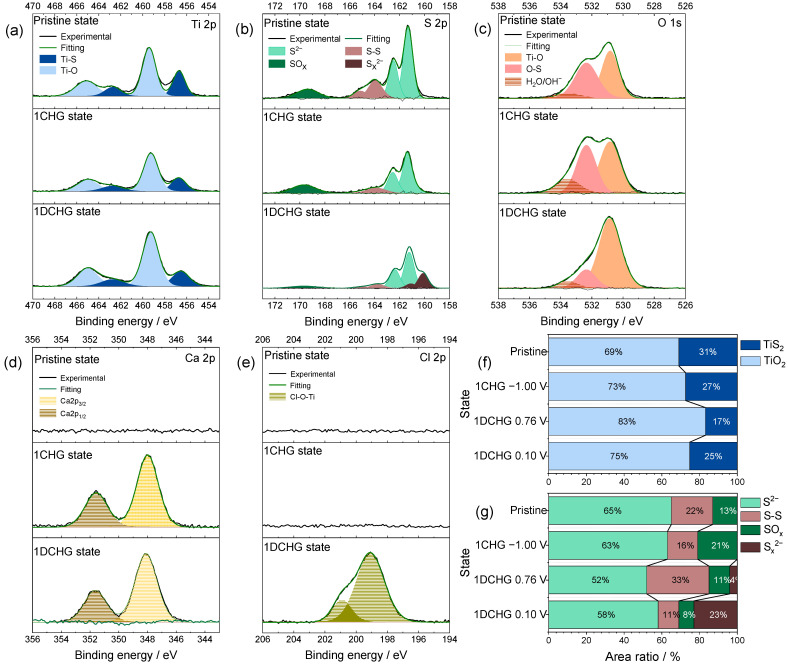
XPS results of surface species on TiS_2_ electrodes cycled in 8.0 M CaCl_2_ within the practical potential window (−1.00 to 0.10 V): (**a**) Ti 2p, (**b**) S 2p, (**c**) O 1s, (**d**) Ca 2p, and (**e**) Cl 2p. Quantitative comparison of the peak area ratios for (**f**) Ti 2p and (**g**) S 2p to demonstrate the suppression of oxidative surface products within the practical potential window and the dynamic interfacial equilibrium among Ti–S, CaS, and SO_x_ species.

The surface composition and chemical states of TiS_2_ electrodes cycled in 8 M CaCl_2_ were examined by XPS to probe interfacial reactions. As shown in Figure 7a,b, the Ti 2p and S 2p spectra evolved systematically with potential. After the first charge (−1.00 V), the intensities of the TiS_2_- and S^2−^-related peaks decreased, whereas the signals associated with TiO_2_ and S–O (SO_x_) species intensified, consistent with residual oxidation products rather than the formation of new species at the reducing potential. This trend is consistent with the partial oxidation during the preceding anodic (positive potential) process, with subsequent Ca^2+^ intercalation not fully reversing the surface species. Upon discharge, the polysulfide (S_z_^2−^, 2 ≤ z ≤ 8) features reappeared, whereas the S_8_ signal diminished, indicating partially reversible sulfur-centered redox within the range −1.00 to 0.10 V. These observations support dynamic surface reorganization rather than formation of a permanently passivating layer. In the Cl 2p region, no additional component attributable to a distinct Ti–Cl phase was resolved; instead, the Cl signal is dominated by CaCl_2_-like environments originating from the bulk electrolyte and Ca–Cl-containing interphase species, consistent with chloride participating in the SEI/interphase without forming a separate, spectroscopically resolvable titanium–chloride compound. This surface-localized character also rationalizes why no distinct TiO_2_ or S_8_ reflections are resolved in the ex situ XRD patterns (Section 2.4). In this context, the ex situ Ti 2p spectra collected after the first charge/discharge are expected to be dominated by conversion products and surface reconstruction, and only a limited, short-lived fraction of Ti^3+^ is anticipated; this helps to explain why the Ti 2p envelope exhibits only subtle changes rather than a clearly resolved Ti^3+^ doublet, even though the CV indicates a small Ti-centered contribution in the −0.48/−0.24 V couple within the restricted potential window [33,43]. In the present study, Raman spectroscopy was applied to the pristine TiS_2_ electrode (Figure 3b) and to the bulk CaCl_2_ electrolytes to probe concentration-dependent solvation-structure changes (Figure 2), whereas the speciation of CaS and polysulfides on cycled electrodes is primarily resolved by potential-resolved XPS and electrochemical signatures; operando or carefully optimized ex situ Raman measurements on cycled TiS_2_ electrodes therefore remain an important direction for future work.

Quantitative deconvolution (Figure 7f,g) showed that the oxide-related fraction for Ti increased modestly (~6%) within −1.00 to 0.10 V and increased further when the window was extended to 0.76 V (−1.00 to 0.76 V), while the S–S fraction increased from ~8% to ~22% under the same comparison. These differences indicate that the practical potential range helps suppress TiS_2_ → TiO_2_ conversion and S_8_ formation, thus preserving the intrinsic Ti–S bonding network more effectively. The O 1s and Ca 2p spectra (Figure 7c,d) displayed Ti–O (~530.9 eV) and O–S (~532.3 eV) features, along with a ~533.6 eV component commonly attributed to adsorbed H_2_O/OH^−^. After charging (−1.00 V, reductive state), the enhanced hydroxide-related signal and Ca 2p feature near ~347.9 eV were consistent with residual Ca(OH)_2_ from the prior oxidative step rather than new formation at negative potentials [44]. In combination, these signatures are consistent with a thin, self-limiting interphase that limits water access and are consistent with a reduced contribution from HER-type parasitic reactions in the concentrated electrolyte. In concentrated media with low free water activity, the electrochemical response is consistent with an interphase that prevents the passage of electrons yet allows the permeation of ions to stabilize the interface [32].

During subsequent discharge, a slightly higher binding-energy component (~348.1 eV) could indicate the formation of sulfate-containing species such as CaSO_4_ [45,46]. However, this assignment is tentative because the ~0.2 eV shift is within the typical instrumental/charge-reference uncertainty. The Cl 2p spectrum (Figure 7e) shows the absence of detectable chlorine-derived solids after charging within the XPS detection limits of this study; under these conditions, dominant anion-derived passivation appears unlikely. The weak component near ~199.2 eV could arise from reversible Cl^−^ adsorption on oxidized Ti sites, or alternatively from trace residual CaCl_2_ adsorbates with similar binding-energy signatures. Overall, the surface chemistry of TiS_2_ was consistent with the presence and partial reversibility of CaS and polysulfide species, together with limited Ca(OH)_2_ and CaSO_4_ generation. High salt concentrations may stabilize these species by reducing the hydrolysis and lowering the water activity, thereby mitigating the irreversible formation of TiO_2_ and S_8_, which supports a working model of dynamic interfacial equilibrium under concentrated conditions [47,48].

### 2.6. Proposed Reaction Mechanism and Correlation Framework

**Figure 8:**
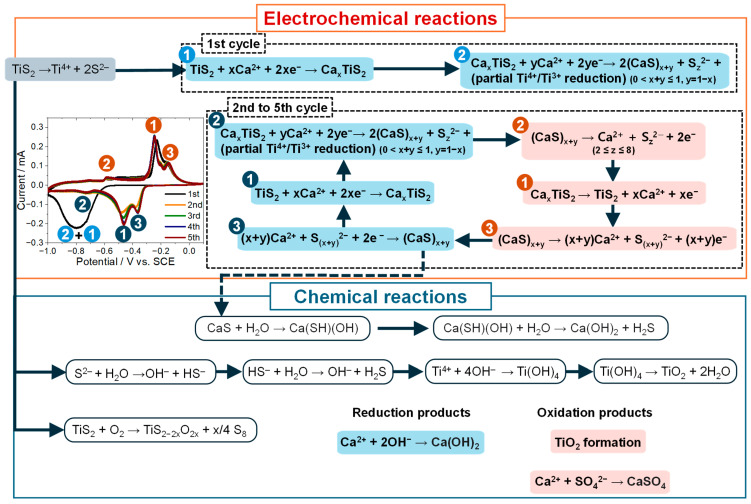
Schematic illustration of the proposed electrochemical and chemical reaction mechanisms of TiS_2_ in 8.0 M CaCl_2_ electrolyte. The diagram distinguishes the activation in the 1st cycle from the 2nd-to-5th-cycle region, which represents the representative steady regime shown in the inset CV. The formation of metallic Ti is excluded; charge compensation occurs through a partial Ti(IV)/Ti(III) redox reaction coupled with the CaS ↔ S_z_^2−^ (polysulfide) equilibrium. Chemical side reactions such as CaS hydrolysis → Ca(OH)_2_ and limited CaSO_4_ formation are included as interfacial processes that modulate the relative contribution of HER and the overall interfacial stability.

Based on the potential-resolved electrochemistry (CV), lattice responses (XRD), and interfacial speciation (XPS), a working mechanism was developed to describe the intercalation/deintercalation of Ca^2+^ in TiS_2_ in concentrated CaCl_2_ electrolytes. As illustrated in Figure 8, the overall process is consistent with the coupling between the intercalation and conversion reactions, modulated by both the potential and electrolyte concentration. During the first charge, Ca^2+^ was intercalated into the TiS_2_ lattice to form Ca_x_TiS_2_. With further reduction, Ca^2+^ interacted with sulfur by cleaving S–S bonds and yielding CaS together with polysulfide species (S_z_^2−^) at or near the interface [49,50]. Charge compensation is consistent with partial reduction of Ti(IV) to Ti(III). These steps are consistent with the injection of electrons into Ti–S bonds, associated bond polarization, and lattice expansion. Minor Ca(OH)_2_ signals are more parsimoniously attributed to the prior positive-potential step and/or ex situ exposure, rather than in situ hydrolysis of CaS under reducing conditions. Under these conditions, the resulting reaction-derived layer is consistent with lower HER activity, plausibly by limiting water access while permitting ion transport.

Upon discharge, CaS underwent partial oxidation that partially regenerated TiS_2_ and released Ca^2+^ together with polysulfide (S_z_^2−^) intermediates into the electrolyte. The CaS ↔ S_z_^2−^ interconversion proceeded with partial reversibility, while charge balance appears to be provided primarily by the Ti(IV)/Ti(III) couple. From the second cycle onward, this CaS/S_z_^2−^ equilibrium appears to increasingly govern charge compensation; within the XRD/XPS detection limits, metallic Ti signatures were not observed. Consistent with this assignment, ex situ XRD showed that the (001) reflection shifted upon charge and recovered with hysteresis on discharge (Figure 6b), consistent with interlayer expansion and contraction; the incomplete return to the initial position evidences residual disorder, including partial amorphization and defect formation (e.g., sulfur vacancies) [43,51]. These defect signatures are consistent with changes in local electronic transport and may provide limited redox buffering that helps stabilize the CaS/polysulfide couple.

The correlation framework (Figure 8) delineates three potential regimes: (i) −1.00 to −0.60 V, characterized by dominant sulfur reduction to form CaS and S_z_^2−^ intermediates without detectable metallic Ti; (ii) −0.60 to −0.20 V, the Ti(IV)/Ti(III) redox coupled with reversible lattice breathing; and (iii) above 0 V, over-oxidation to TiO_2_ and S_8_, which were minimized within the practical window. Under these conditions, stable electrochemical behavior is consistent with a cooperative balance among controlled potential, defect-mediated pathways, and interfacial stabilization by Ca(OH)_2_ and CaSO_4_.

The analysis relies on structural/spectroscopic and electrochemical indicators of Ca^2+^ intercalation/deintercalation. The absolute bulk Ca stoichiometry was not quantified in this study, and the claims advanced here are based on the convergence of CV, XRD, and XPS signatures. Operando techniques, such as differential electrochemical mass spectrometry, gas chromatography, inductively coupled plasma optical emission spectroscopy, and time-of-flight secondary ion mass spectrometry, could further resolve the kinetic parameters underlying this mechanism. Overall, this framework links the electrolyte concentration, interfacial chemistry, and structural reversibility in TiS_2_, thus indicating the extent to which concentrated electrolytes can promote a dynamic interfacial equilibrium that supports multivalent-ion storage under the tested conditions. In addition, combining the present structure–electrochemistry analysis with ICP-based operando or ex situ quantification of dissolved Ca, Ti, and S species would be valuable for assessing dissolution, chemical side reactions, and overall mass balance in concentrated CaCl_2_ electrolytes.

## 3. Materials and Methods

### 3.1. Electrolyte Preparation

Calcium chloride dihydrate (CaCl_2_·2H_2_O, 99.0–105.0%, Alfa Aesar, Haverhill, MA, USA) was dissolved in high-purity water (Burdick & Jackson, HPLC grade, Muskegon, MI, USA) to prepare aqueous CaCl_2_ electrolytes at defined concentrations. The concentrations of the prepared solutions were 1.0, 4.0, 7.0, and 8.0 M. Solutions were prepared gravimetrically and brought to volume at 25 ± 2 °C to define molarity. The maximum concentration investigated was 8.0 M, which we selected as a practical upper limit under our preparation conditions at 25 ± 2 °C; at higher nominal concentrations, the markedly increased viscosity and poor cell wetting prevented homogeneous mixing and reliable electrochemical testing, so concentrations above 8.0 M were not investigated further in this study. For consistency with the text, 1.0 and 4.0 M were designated as dilute electrolytes, and 7.0 and 8.0 M were designated as concentrated electrolytes. Each solution was magnetically stirred (2 h, ~500 rpm) to ensure complete dissolution and stored in tightly sealed polypropylene bottles prior to electrochemical and spectroscopic measurements.

### 3.2. Electrochemical Measurements

All electrochemical measurements were conducted using a battery testing system (WBCS 3000, WonATech, Seoul, Republic of Korea) in a three-electrode glass cell at 25 ± 2 °C. The TiS_2_ electrode served as the working electrode, an activated carbon electrode served as the counter electrode, and an SCE (RE-2BP, Qrins, 3.3 M KCl, Seoul, Republic of Korea) was used as the reference electrode. The TiS_2_ composite electrode was fabricated using titanium disulfide (TiS_2_, 99.9%, −200 mesh; Sigma-Aldrich, St. Louis, MO, USA) as the active material, Super P carbon black (99+ %; Alfa Aesar, Haverhill, MA, USA), graphite powder (SNO-15; used as received), and poly (vinylidene fluoride) (PVdF, Mw ~ 5.34 × 10^5^; Sigma-Aldrich, St. Louis, MO, USA). The powders were mixed in a mass ratio of 80:9:2:9 (TiS_2_:Super P:graphite:PVdF) in N-methyl-2-pyrrolidone (NMP, 99+ %; JUNSEI, Tokyo, Japan) to form a homogeneous slurry, coated on titanium foil (99.5%; Nilaco, Tokyo, Japan), and dried under vacuum (80 °C, 12 h). The resulting electrode had a mass loading of ~2 mg cm^−2^ and a film thickness of ~30 μm. The counter electrode was prepared from activated carbon powder (−100 mesh; Sigma-Aldrich, St. Louis, MO, USA) and PVdF at a mass ratio of 9:1 using the same solvent and drying procedure. All electrodes were stored in a desiccator prior to assembly. The prepared CaCl_2_ electrolytes (1.0, 4.0, 7.0, and 8.0 M) were used as the ionic media. Cyclic voltammetry was performed at a scan rate of 0.1 mV s^−1^ within the following potential windows: −1.00 to 0.76 V, −1.00 to 0.10 V, −0.40 to 0.76 V, −0.60 to 0.76 V, −0.50 to 0.10 V, −1.00 to −0.28 V, −1.00 to −0.40 V, and −1.00 to −0.17 V (all vs. SCE). Galvanostatic charge–discharge tests were performed in −1.00 to 0.76 V and −1.00 to 0.10 V at a C-rate of 0.1 C. Before each electrochemical measurement, the cell was rested for 5 h to allow it to approach equilibrium, and all tests were conducted at 25 ± 2 °C under quiescent conditions. In this study, the term practical potential window refers to the potential interval that yields a reproducible capacity without large irreversible voltammetric peaks or visible gas evolution under the test conditions.

### 3.3. Spectroscopic and Structural Analysis

Electrolytes of different concentrations were analyzed using Raman spectroscopy (LabRAM HR-800, Horiba Jobin Yvon, Tokyo, Japan) to examine the evolution of the solvation structure. Spectra were recorded in the O–H stretching region (2800–4000 cm^−1^) using a 514 nm excitation laser at 25 ± 2 °C. Raman spectroscopy was chosen because the O–H stretching region is highly sensitive to hydrogen-bonding and ion-pairing environments and can be measured under the same conditions as the electrode samples, thereby providing solvation descriptors at the predefined CaCl_2_ concentrations used in the electrochemical tests. The TiS_2_ electrodes were characterized ex situ by Raman spectroscopy, XRD, and XPS before and after electrochemical cycling. Raman spectra in the 100–900 cm^−1^ range were collected to monitor vibrational modes associated with the Ti–S lattice and sulfur species. XRD measurements (MiniFlex 600, Rigaku, Tokyo, Japan) were performed over 2θ = 10–80° at a scan rate of 1° min^−1^ at 25 ± 2 °C. All the XRD measurements were conducted at the Advanced Energy and Display Materials Analysis Center of Soonchunhyang University using equipment registered under the identifier NFEC-2018-12-247471. Surface compositions were examined using XPS (AXIS Nova, Kratos Analytical, Manchester, UK). Survey and high-resolution spectra were first acquired without Ar^+^ sputtering; gentle Ar^+^ sputtering was used only for robustness/depth checks, and the compositional assignments in the main text were based on the unsputtered spectra. The binding energies were referenced to C 1s (284.8 eV) and the spectra were deconvoluted using Thermo Avantage software (Thermo Fisher Scientific, version 5.9931, Seoul, Republic of Korea).

## 4. Conclusions

The interfacial and structural behavior of TiS_2_ in concentrated aqueous CaCl_2_ electrolytes was systematically examined to elucidate the mechanism of Ca^2+^ intercalation/deintercalation. An increase in the CaCl_2_ concentration from 1.0 to 8.0 M was accompanied by a qualitatively decreased HER contribution, as inferred from reduced gas evolution and improved coulombic efficiency, and an expanded practical potential window. Stepwise CV identified the potential range of −1.00 to 0.10 V (vs. SCE) as a practical operating window that minimized TiO_2_/S_8_ formation while preserving the Ca^2+^-intercalation signatures. Ex situ XRD revealed reversible interlayer expansion/contraction; peak broadening consistent with partial amorphization and defect formation (e.g., sulfur vacancies) suggested modified transport pathways and interfacial charge-transfer characteristics. XPS indicated CaS and polysulfides (S_z_^2−^) as the prevalent interfacial species, along with limited Ca(OH)_2_ and CaSO_4_ at the interface. Under these conditions, the observed species are consistent with reduced water decomposition and improved interfacial stability. Raman spectra distinguished dilute (1.0–4.0 M) from concentrated (7.0–8.0 M) CaCl_2_ by redistribution and blue-shifted O–H features, consistent with lower free-water activity and enhanced Ca–Cl association at higher concentrations. Together with XPS-identified CaS/polysulfide-rich interphases (limited Ca(OH)_2_/CaSO_4_ and no Cl-containing reduction products within XPS detection limits), these concentration-dependent descriptors rationalize the sustained reversible Ca^2+^ intercalation within the operational window under concentrated conditions. Collectively, the results support a correlation framework linking the electrolyte concentration, potential window, interfacial chemistry, and structural reversibility, suggesting that stable behavior is associated with a controlled potential window and defect signatures indicative of partially amorphous states. This study relied on ex situ characterization and the observed trends suggest the need for future operando and long-term evaluations to further refine the mechanism.

## Figures and Tables

**Table 1:** Interlayer spacing of TiS_2_ electrodes calculated from the respective (001) reflection using Bragg’s law, demonstrating reversible recovery consistent with the pseudoelastic response of the layered lattice during Ca^2+^ intercalation and deintercalation.

State	Miller Index/(hkl)	Degree/2θ	Interlayer Spacing/Å
Pristine	(001)	15.56	5.69
1CHG	(001)	15.34	5.77
1DCHG	(001)	15.52	5.71
2CHG	(001)	15.42	5.74
2DCHG	(001)	15.52	5.71

## Data Availability

The original contributions presented in this study are included in the article/supplementary material. Further inquiries can be directed to the corresponding author.

## Supplementary Materials

The following supporting information can be downloaded at: https://www.mdpi.com/article/10.3390/ijms262411971/s1.
