# Supplementary material for: Mechanism-Informed Interfacial Chemistry and Structural Evolution of TiS2 During Ca2+ Intercalation in Concentrated Aqueous CaCl2 Electrolytes
Source: Int J Mol Sci. 2025 Dec 12;26(24):11971. doi: 10.3390/ijms262411971 (PMC12733094; doi:10.3390/ijms262411971)
Supplement: Supplementary file 1 [file ijms-26-11971-s001.zip › ijms-3993255-supplementary.docx]

Supporting Information

Mechanism-Informed Interfacial Chemistry and Structural Evolution of TiS_2_ during Ca^2+^ Intercalation in Concentrated Aqueous CaCl_2_ Electrolytes

SangYup Lee ^1^, Sujin Seong ^1^, Seunga Yang ^1^, and Soon-Ki Jeong ^1,2,3,^*

^1^ Department of Future Convergence Technology, Graduate School, Soonchunhyang University,
Soonchunhyang-ro 22-gil, Sinchang-myeon, Asan-si 31538, Chungcheongnam-do, Republic of Korea; tmddk1107@sch.ac.kr (S.L.); sujin@sch.ac.kr (S.S.); 20237450@sch.ac.kr (S.Y.)

^2^ Department of Energy Engineering, Soonchunhyang University, Soonchunhyang-ro 22-gil,
Sinchang-myeon, Asan-si 31538, Chungcheongnam-do, Republic of Korea

^3^ Advanced Energy Research Center, Soonchunhyang University, Soonchunhyang-ro 22-gil,
Sinchang-myeon, Asan-si 31538, Chungcheongnam-do, Republic of Korea

***** Correspondence: hamin611@sch.ac.kr (S.-K.J.)

*Corresponding Author

*E-mail address: hamin611@sch.ac.kr (S.-K. Jeong)*


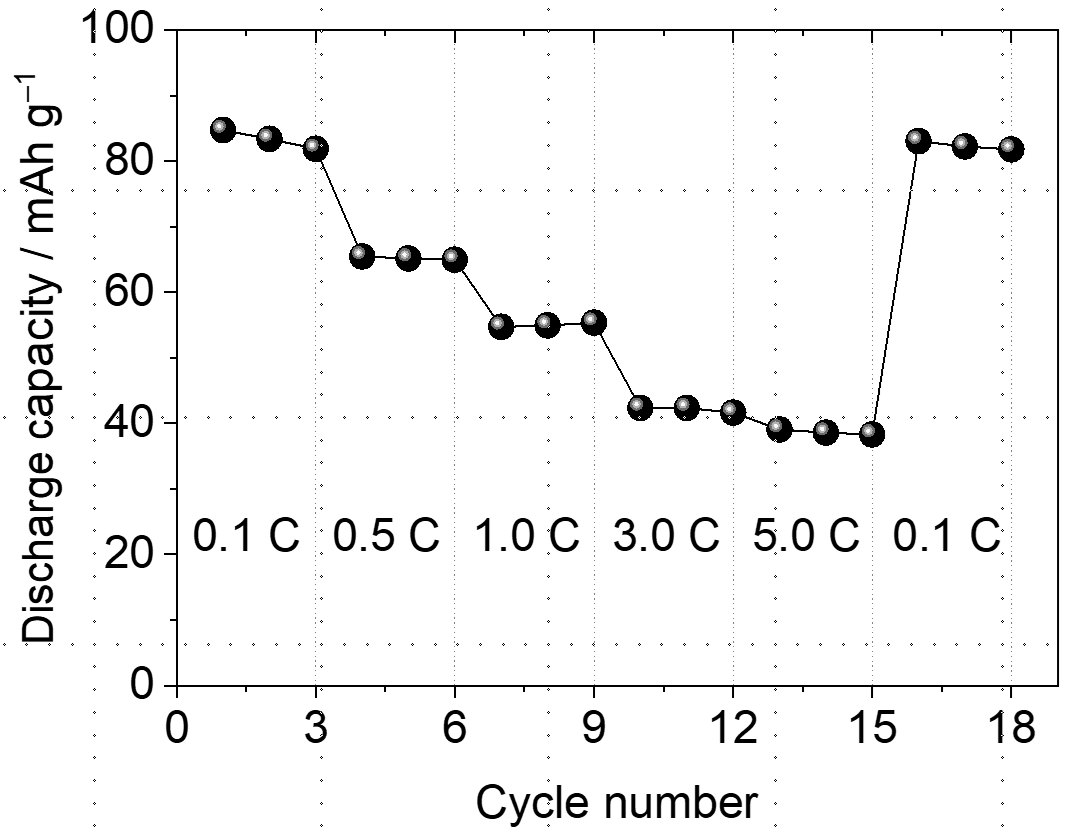


**Figure S1.** Electrochemical performance of the TiS_2_ electrode in 8.0 M CaCl_2_ within the practical potential window (−1.00 to 0.10 V): rate capability.


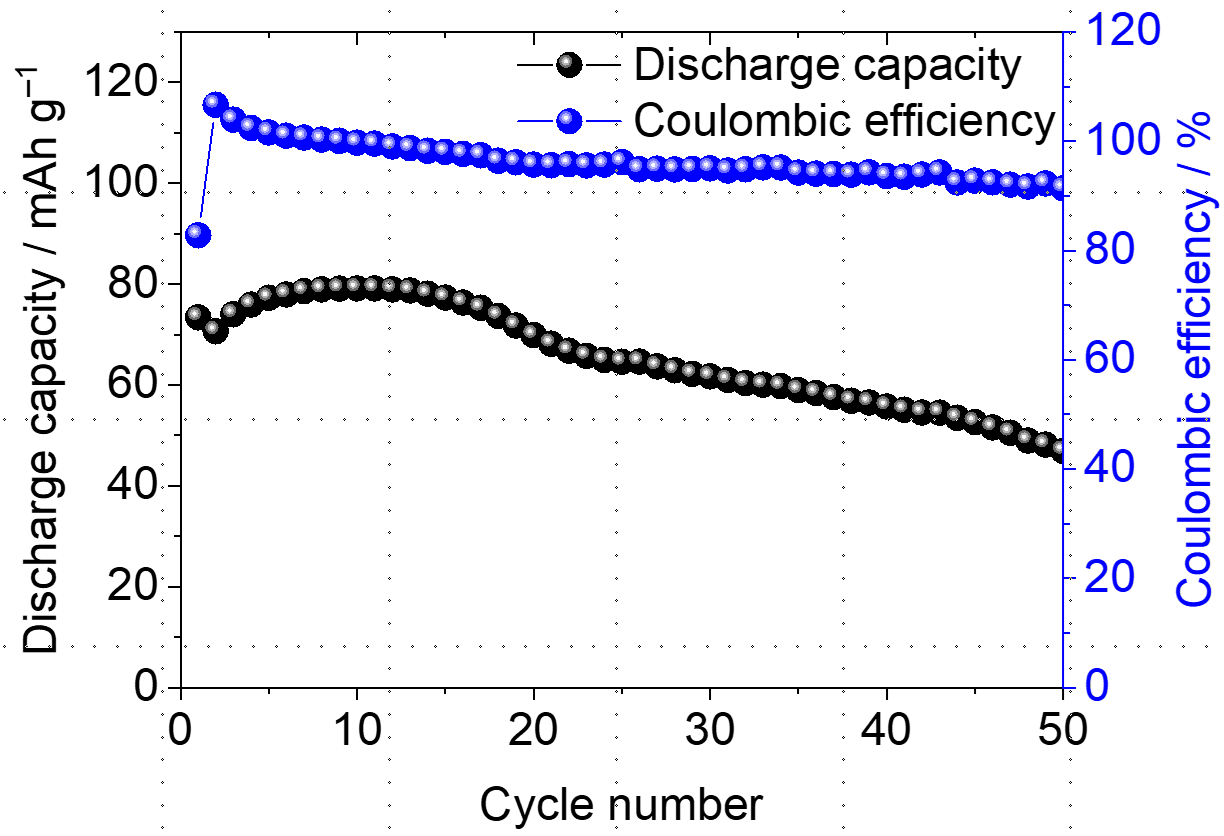


**Figure S2.** Electrochemical performance of the TiS_2_ electrode in 8.0 M CaCl_2_ within the practical potential window (−1.00 to 0.10 V): cycling performance results at 0.1 C.

**Table S1.** Normalized area ratiosA_3200_/A_3420_ and A_3600_/A_3420_ for the deconvoluted O–H stretching components as a function of CaCl₂ concentration.

| **CaCl_2_ concentration / M** | **A_3200_/A_3420_** | **A_3600_/A_3420_** |
| --- | --- | --- |
| 0 (pure water) | 0.634 | 0.174 |
| 1.0 | 0.424 | 0.085 |
| 4.0 | 0.271 | 0.080 |
| 7.0 | 0.184 | 0.050 |
| 8.0 | 0.157 | 0.040 |
